# Topological metastability supported by thermal fluctuation upon formation of chiral soliton lattice in $\hbox {CrNb}_3\hbox {S}_6$

**DOI:** 10.1038/s41598-020-74945-6

**Published:** 2020-10-29

**Authors:** T. Honda, Y. Yamasaki, H. Nakao, Y. Murakami, T. Ogura, Y. Kousaka, J. Akimitsu

**Affiliations:** 1 Institute of Materials Structure Science, High Energy Accelerator Research Organization (KEK), Tsukuba, 305-0801 Japan; 2 Research and Services Division of Materials Data and Integrated System (MaDIS), National Institute for Materials Science (NIMS), Tsukuba, 305-0047 Japan; 3 Center for Emergent Matter Science (CEMS), RIKEN, Wako, 351-0198 Japan; 4 PRESTO, Japan Science and Technology Agency (JST), Saitama, Japan; 5 Department of Physics and Mathematics, Aoyama-Gakuin University, Sagamihara, Kanagawa 252-5258 Japan; 6 Department of Physics and Electronics, Osaka Prefecture University, Osaka, 599-8531 Japan; 7 Research Institute for Interdisciplinary Science, Okayama University, Okayama, 700-8530 Japan

**Keywords:** Magnetic properties and materials, Topological defects

## Abstract

Topological magnetic structure possesses topological stability characteristics that make it robust against disturbances which are a big advantage for data processing or storage devices of spintronics; nonetheless, such characteristics have been rarely clarified. This paper focused on the formation of chiral soliton lattice (CSL), a one-dimensional topological magnetic structure, and provides a discussion of its topological stability and influence of thermal fluctuation. Herein, CSL responses against change of temperature and applied magnetic field were investigated via small-angle resonant soft X-ray scattering in chromium niobium sulfide ($\hbox {CrNb}_3\hbox {S}_6$). CSL transformation relative to the applied magnetic field demonstrated a clear agreement with the theoretical prediction of the sine-Gordon model. Further, there were apparent differences in the process of chiral soliton creation and annihilation, discussed from the viewpoint of competing between thermal fluctuation and the topological metastability.

## Introduction

**Figure 1:**
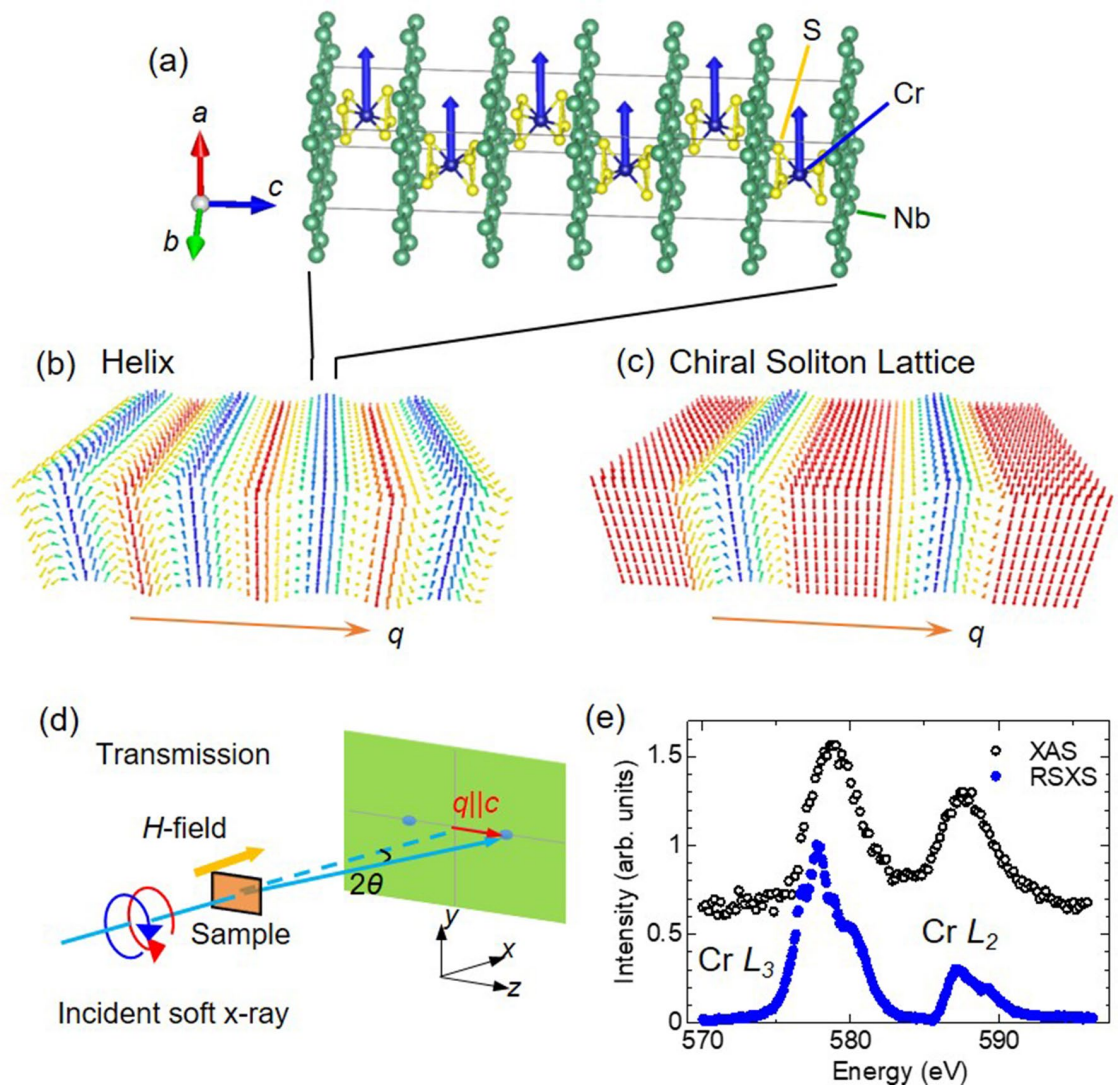
(**a**) Crystal structure of $\hbox {CrNb}_3\hbox {S}_6$ and (**b**,**c**) schematic drawings of magnetic structures of helix and chiral soliton lattice (CSL), respectively. (**d**) Experimental setup for small-angle resonant soft X-ray scattering (RSXS). (**e**) Energy spectra for X-ray absorption (XAS) and RSXS. Spectral data is vertically shifted for clarity.

Magnets with chiral crystal structure provide a good platform for exploring non-trivial spin textures due to Dzyaloshinskii-Moriya (DM) interaction which comes from the spin-orbit interaction and the lack of inversion symmetry of crystals. In these years, spin textures with topological features in the chiral magnets have been intensively investigated because of their promising potential for developing novel spintronics devices. For example, skyrmions, topological magnetic structures, show a triangle crystallization of the stable magnetic whirls that emerge in the 2D or 3D magnetic system^1^. On the other hand, the chiral magnetic configuration in the 1D system has been considered as the chiral magnetic soliton lattice (CSL). The formation of CSL has been experimentally demonstrated in chiral magnets, such as $\hbox {CrNb}_3\hbox {S}_6$^2^ (Fig. 1a), $\hbox {Yb}(\hbox {Ni}_{1-x} \hbox {Cu}_x)_3\hbox {Al}_9$^3^, and so on. The ground state of magnetic structure in the noncentrosymmetric chiral structure is a helical magnetic structure (Fig. 1b), which originates from the competition between the symmetric exchange interaction and the antisymmetric DM interaction $\varvec{D}\cdot (\varvec{S}_i\times \varvec{S}_j)$, where $\varvec{D}$ is the DM vector determined by the symmetry of bonding between localized neighboring spins $\varvec{S}_i$ and $\varvec{S}_j$. An application of a magnetic field perpendicular to the helical axis induces a decrease in the magnetic wave number to form the CSL, which consists of ferromagnetic (FM) domains periodically partitioned by magnetic solitons (kink-like $2\pi$ domain walls), as shown in Fig. 1c^4,5^, and finally, for a continuous magnetic transition from the CSL to the forced ferromagnetic (FFM) state.

In this study, we consider the classical magnetic moment, $m = m_q (\cos \phi (z), \sin \phi (z), 0)$ with $\phi (z)$ being the phase angle on the position *z* along the helical axis (*z* axis). The helix is caused by the isotropic FM interaction (*J*) and the anisotropic DM interaction ($D = |\varvec{D}|$), and its pitch angle is given by $\tan ^{-1}(D/J)$. The formation of CSL is derived from the phenomenogical Ginzburg-Landau (GL) free energy function,

1 $$\begin{aligned} F=\frac{J^\prime m_q^2}{L}\int _0^L\left\{ \frac{1}{2}(\partial _z \phi )^2 -q_0\partial _z\phi -b\cos \phi \right\} dz, \end{aligned}$$

with *L* being the distance between the chiral magnetic solitons, $J^\prime = (J^2 + D^2)^{1/2}$, $q_0 = D/J$ and $b = \tilde{H}/Jm_q$. The phase angle of magnetic moment in the CSL state is given by $\cos \phi (z) = 2\text {sn}^2(\gamma z)-1$ with $\gamma =\pi q_0/4E(\kappa )$^4^. Here, sn is the Jacobian elliptic function and $E(\kappa )$ is the complete elliptic integral of the second kind with $\kappa$ being the elliptic modulus^6^. The Fourier transformation of these magnetic configurations gives rise to the magnetic modulation vector $q_{CSL} = (0, 0, q)$ with

2 $$\begin{aligned} q=\frac{\gamma \pi }{K(\kappa )}=\frac{\pi ^2q_0}{4K(\kappa )E(\kappa )}, \end{aligned}$$

where $K(\kappa )$ is the complete elliptic integral of the first kind. Minimizing the GL free energy gives the $\kappa$-dependence of magnetic fields, $H/H_c = [\kappa /E(\kappa )]^2$, and thus, the critical field of the CSL-FFM transition, corresponding to $\kappa =1$, is given by $H_c = Jm_q (\pi q_0/4)^2$.

These magnetic field response has experimentally been observed in chromium niobium sulfide $\hbox {CrNb}_3\hbox {S}_6$^7^. The material has a layered hexagonal structure with the chiral space group $P6_322$, as shown in Fig. 1a. The intercalated chromium atoms occupy the octahedral interstitial holes between the trigonal prismatic layers of 2H-$\hbox {NbS}_2$. The trivalent Cr ion has localized electrons ($3d^3$) and spins of $S = 3/2$. Breaking inversion symmetry of the chiral crystal induces the DM vector aligning parallel to the crystallographic *c* axis. The Cr spins are arranged ferromagnetically within the *ab* plane and are modulated along the *c* axis^8^. At zero magnetic field, the ground state of magnetic structure is the helical magnetic structure with the magnetic modulation wave-length of $\sim 48\ \hbox {nm}$ below $T_{\mathrm{N}} = 120\ \hbox {K}$, and the CSL is formed by the application of magnetic fields. The magnetic-field dependence of lattice constant of CSL observed by the Lorentz-type transmission electron microscopy shows a good agreement with that estimated from Eq. (2)^7^. A negative magnetoresistance effect due to the interaction between the localized electrons of Cr ion and the itinerant electrons of Nb ions was detected in the vicinity of CSL formation from the FFM phase^9^. The creation/annihilation of a single chiral soliton has also been detected via magnetoresistance and magnetization measurements^10,11^.

In the present paper, the formation process of the CSL in $\hbox {CrNb}_3\hbox {S}_6$ is investigated via magnetic-field dependence of the higher harmonic magnetic reflections observed by small-angle resonant soft X-ray scattering (RSXS). The diffraction results would make it possible to elucidate in detail the magnetic structure of chiral solitons and its response to changes in temperature and applied magnetic field. Especially, we focus on the influence of thermal fluctuation which should compete with the topological stability on the formation of chiral solitons.

## Results

**Figure 2:**
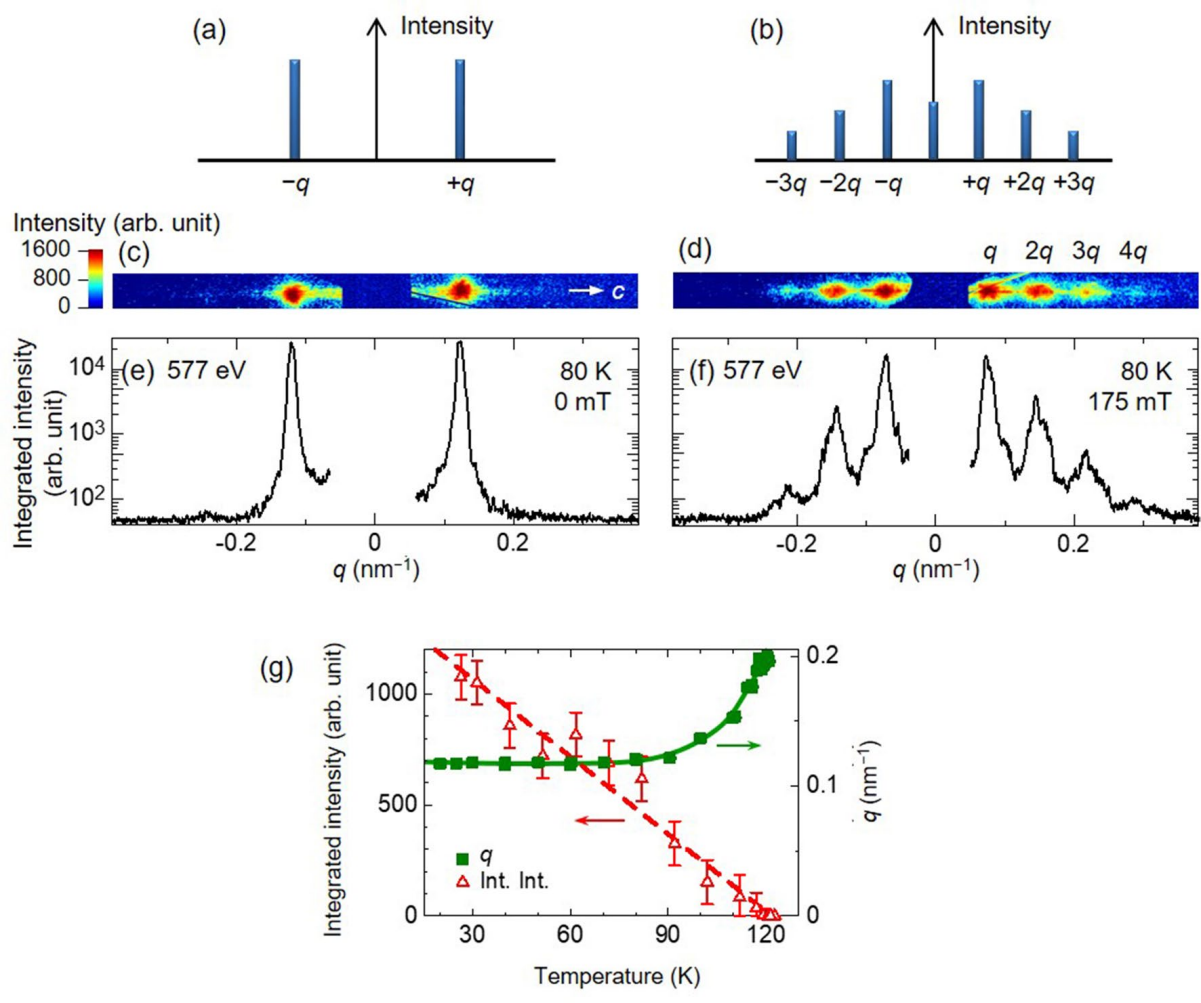
Schematics of magnetic scattering profiles, CCD images [log(log) scale], and the line profiles of small-angle RSXS for helical (**a**,**c**,**e**) and CSL (**b**,**d**,**f**) states, respectively. (**g**) Temperature dependence of the integrated intensity and magnetic modulation vector *q* for magnetic scattering from the helix structure.

We measured the magnetic-field dependence of the magnetic reflections via the small-angle RSXS at Cr $L_3$ absorption edge (Fig. 1e). The RSXS at $L_{2,3}$-edge absorption of 3*d* transition metal possesses high sensitivity to the 3*d* electronic state and thus, is an effective method for detecting of magnetic reflections^12^. The intensities of magnetic reflections are proportional to the absolute square of the magnetic structural factor, which is calculated from the Fourier transformation of the magnetic spatial structure. Subsequently, only the fundamental reflection would be observed in helical structure where the spins align with linearly modulating rotation angle (Fig. 2a). On the contrary, higher harmonic magnetic reflections should emerge in the CSL structure due to the modulation of non-linear spin rotation angles (Fig. 2b). Therefore, the behavior of higher harmonic magnetic reflections directly reflects the spin structure of the CSL.

Small-angle RSXS signals measured at 0 and at 175 mT on the CCD camera with the circularly polarized soft X-ray of 577 eV when $T=80\ \hbox {K}$ are displayed in Fig. 2c,d, respectively, with those line profiles being in Fig. 2e,f. In the absence of magnetic field, only the magnetic fundamental reflections could be observed at $q = \pm 0.121\ \text {nm}^{-1}$ at 80 K, which corresponds to the helix magnetic structure. Magnetic scattering appeared below 121 K and the integrated intensity increased linearly, while the *q* vector gradually decreased as lowering temperature and converged below 90 K as shown in Fig. 2g. With the application of magnetic field, the *q* positions of the magnetic fundamental reflection were displaced to approach the center, and additional magnetic peaks emerged at the higher *q*-region. Owing to the high sensitivity of RSXS method, we were able to observe weak magnetic reflections of higher *q*-region ($q = \pm 0.073$, $\pm 0.145$, $\pm 0.217$ and $0.287\ \text {nm}^{-1}$) with applied magnetic field of 175 mT. Those additional magnetic diffraction spots could be ascribed to the higher harmonics (the second, third, and fourth) magnetic reflections originating from the formation of CSL.

**Figure 3:**
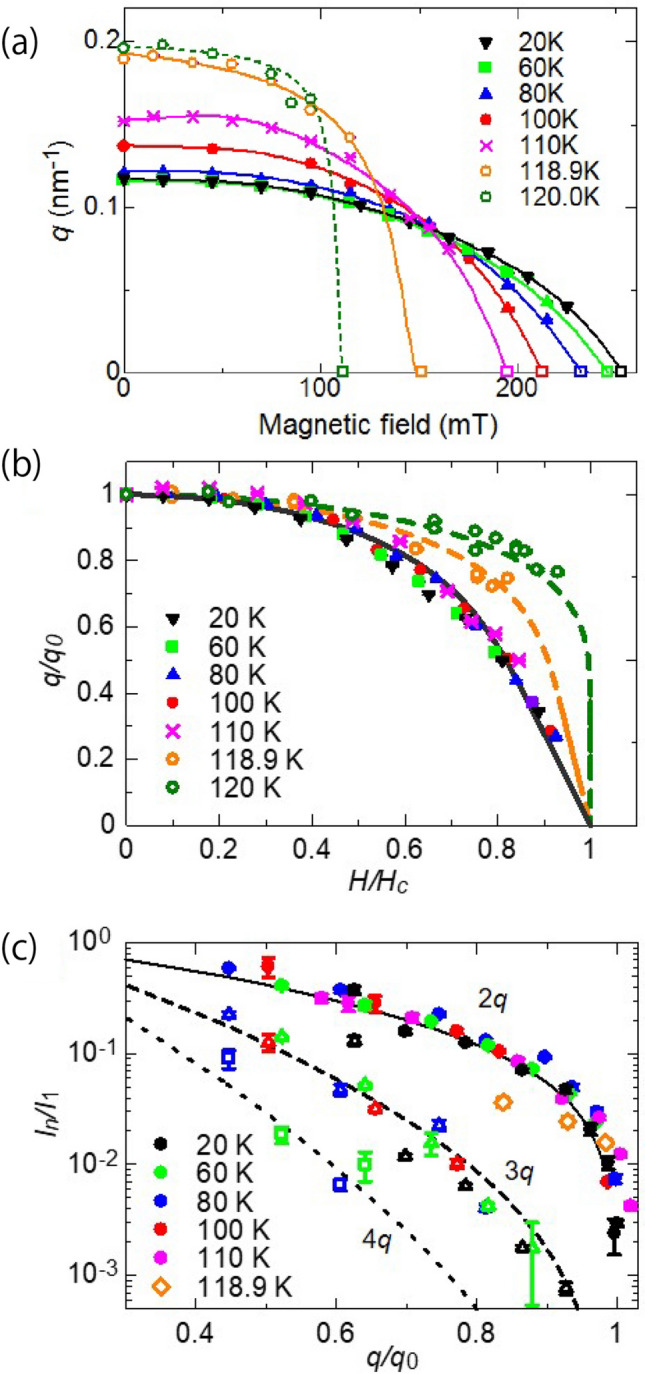
Magnetic-field dependence of chiral magnetic soliton lattice. (**a**) Magnetic field dependence of magnetic propagation wave vector measured at several temperatures, and (**b**) its re-plot data with normalized propagation vector $q/q_0$ of the fundamental magnetic diffraction dependence on normalized magnetic fields $H/H_c$. Here, $q_0$ is the magnetic propagation vector at the zero magnetic field and $H_c$ is the critical magnetic field. Observed data below 110 K show good agreement with the theoretical curve (black solid line), which is corrected by the demagnetization factor. By contrast, data above 110 K (orange and green open circles) deviate from the theoretical curve. Orange and green broke lines are guide to the eyes. (**c**) Integrated intensities for higher harmonic diffractions normalized by the intensity of fundamental magnetic diffraction $I_1$ are plotted against normalized propagation vector $q/q_0$. Closed circle, triangle, and square symbols represent the normalized intensities of $I_2$, $I_3$, and $I_4$, respectively. Black solid, dash, and dot lines indicate theoretical curves calculated by $I_n/I_1 \sim \{J_n^x(\kappa )/ J_1^x(\kappa )\}^2$ ($n=2$, 3, and 4), respectively.

Figure 3a shows the magnetic-field dependence of propagation vector *q* measured at several temperatures. Both the magnetic propagation vector at zero magnetic field ($q_0$) and the critical magnetic field ($H_c$), where the spiral magnetic structure transforms to the forced ferromagnetism, shows strong temperature dependence, thus here we show the data with normalized magnetic field ($H/H_c$) and propagation vector ($q/q_0$) in Fig. 3b. The data below 110 K appear to show a common magnetic field dependence, whereas above 110 K deviated from it. It has been shown that the *q* dependence on magnetic field can be well explained by the sine-Gordon model, i.e. Eq (2), in the previous study of the Lorentz-type transmission electron microscopy^7^. However, the *q* dependence in the present study were deviated from the sine-Gordon model even for the data below 110 K. Since the sample used for the measurement was a flake shape, there is a possibility that the magnetic structures were greatly affected by the demagnetizing field. Therefore, we consider a correction for the magnetic field dependence of Eq (2). For a thin plate magnet, the internal magnetic field *H* is written as $H = H_0 - NM$ with constant demagnetizing factors *N*, magnetization *M*, and the applied magnetic field $H_0$^13^. The theoretical magnetization *M* can be also obtained from the sine-Gordon model, hence Eq. (2) can be modified with the demagnetization factor *N* as a correction parameter. As shown for the solid line in Fig. 3b, the measured values below 110 K are in a good agreement with the corrected curve of Eq. (2). The results indicate the sine-Gordon model corrected for the demagnetization effect can well reproduce the magnetic-field dependence of *q* for CSL in $\hbox {CrNb}_3\hbox {S}_6$. In contrast, the data above 110 K still deviated from the theoretical curve, indicating that the magnetic structure would be different from the theoretically explained CSL near the transition temperature.

In the present paper, we also quantitatively considered the magnetic-field dependence of magnetic diffraction intensities. The magnetic form factor $f_m$ of a magnetic ion is proportional to

3 $$\begin{aligned} f_m\propto (\varepsilon ^\prime \times \varepsilon )\cdot \varvec{m}, \end{aligned}$$

where $\varepsilon$ and $\varepsilon ^\prime$ are the incident and scattered soft X-rays polarizations, respectively, and $\varvec{m}$ is a magnetization vector^14^. Thus, magnetic moment projected parallel to the incident soft X-ray, $m_x$ in the present case, should be mainly detected in the small-angle scattering with circular polarization. The *n*-th intensity ($I_n$) of RSXS at $q_n$ can be calculated by the Fourier transformation of theoretical magnetic structure, which is given by

4 $$\begin{aligned} I_n \propto |f_m \cos \theta _n J_n^x(\kappa )|^2, \end{aligned}$$

with $f_m$ and $\theta _n$ being diffraction angles at the *n*-th higher harmonics. Here, $J_n^x$ is the *n*-th component of Fourier transformation of magnetic moments $m_x$, and is expressed by,

5 $$\begin{aligned} J_0^x (\kappa )&=1+\frac{2{E(\kappa )-K(\kappa )}}{\kappa ^2 K(\kappa )}, \end{aligned}$$

6 $$\begin{aligned} J_n^x (\kappa )&=\frac{\pi ^2}{\kappa ^2 K^2 (\kappa )}\frac{n}{\text {sinh} \{n\pi K^\prime (\kappa )/K(\kappa )\}}, \end{aligned}$$

with $$K^\prime (\kappa ) = K((1-\kappa ^2)^{1/2})$$^5^.

As shown in Fig. 3c, we compared the observed magnetic diffraction intensities with the calculated values by showing the 2nd, 3rd, and 4th higher harmonic diffraction intensities normalized by the fundamental diffraction intensity, such as $I_n/I_1$ with $n = 2$, 3, and 4. To avoid the demagnetization effect, the intensities were plotted against the observed magnetic modulation vector ($q/q_0$) instead of the nominal magnetic field. The solid, broken, dot lines indicate the calculated values of $I_n/I_1 \sim \{J_n^x(\kappa )/ J_1^x(\kappa )\}^2$ for $n=2$, 3, 4, respectively, as the function of $q/q_0 = \pi ^2/4K(\kappa )E(\kappa )$. For the 2nd higher harmonic diffraction, the normalized magnetic diffraction intensities below 110 K seem to be in good agreement with the theoretical curve, whereas the data for 118.9 K deviates from it as well as in the case of magnetic propagation vector. The 3rd and 4th magnetic reflections could only be measured at a limited number of temperature and magnetic points because the intensities were too weak and/or the magnetic scattering was out of the measurable range due to the direct beam catcher. As for the measured data, it can be said that the theoretical equations and observed values are in agreement. Those results suggest that the theoretical sine-Gordon model quantitatively reproduced not only the magnetic field dependence of propagation vector but also that of magnetic diffraction intensities for the CSL in $\hbox {CrNb}_3\hbox {S}_6$. Such agreement with the theoretical model for the diffraction intensity of CLS has been also seen for the CSL in $\hbox {Yb}(\hbox {Ni}_{1-x}\hbox {Cu}_x)_3 \hbox {A}_{19}$^3^.

**Figure 4:**
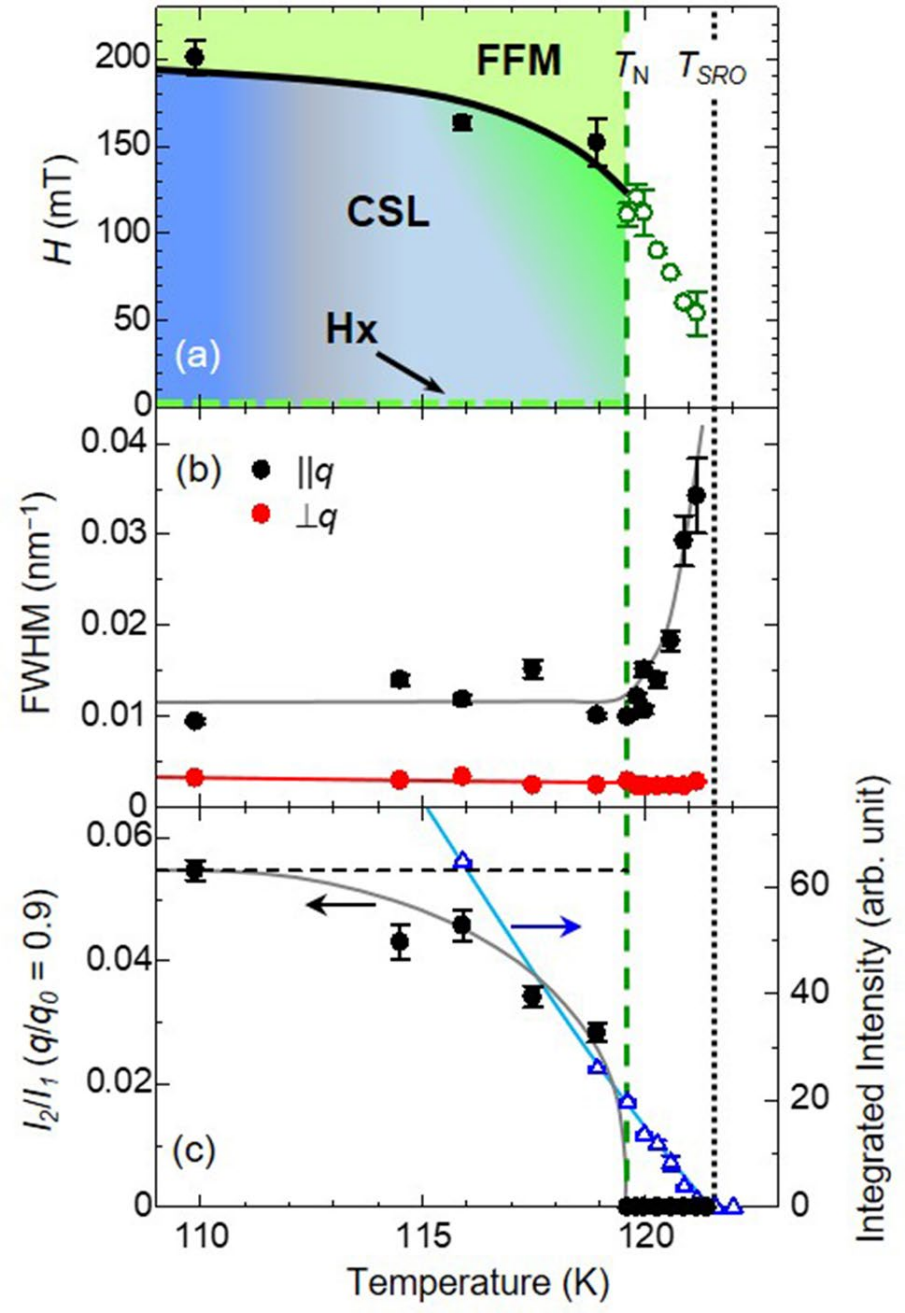
(**a**) The magnetic phase diagram of $\hbox {CrNb}_3\hbox {S}_6$ near magnetic ordering temperature. Black and green symbols denote critical magnetic field $H_c$ where the transitions from the chiral soliton lattice (CSL) or helix (Hx) to the forced ferromagnetic (FFM) occur below and above $T_\text {N} = 119.5$ K, respectively. (**b**) Temperature dependence of the full-width of half maximum (FWHM) for the fundamental magnetic diffraction peak along parallel (||*q*) and perpendicular ($\perp q$) to the magnetic propagation vector *q*, respectively. $T_\text {N}$ is determined by the temperature dependence of the FWHM for ||*q*. (**c**) Temperature dependence of integrated intensity for the fundamental magnetic diffraction ($I_1$) and for $I_2$ normalized by $I_1$ as shown by blue and black symbols, respectively. The solid lines are guides to the eyes. The temperature dependence of $I_2/I_1$ is measured under applied magnetic field where the magnetic propagation vector became $q/q_0=0.9$. Black dash line indicates theoretically estimated intensity of $I_2/I_1$ at $q/q_0 = 0.9$. While $I_2$ is not observable above $T_\text {N}$, $I_1$ can still be detected in the temperature region of $T_\text {N}<T<T_{RSO}$.

Figure 4a represents the magnetic phase diagram near the magnetic ordering temperature determined by the results of small-angle RSXS measurements. This phase diagram of the present study differs from that of the previous studies obtained by magneto-resistance^9^ and magnetization measurements^15–17^, such as the transition temperature $T_N$ and the critical magnetic field $H_c$. For the phase boundary on the magnetic field, it can be ascribed to the difference in the sample shape due to the demagnetization effect as discussed above. The transition temperature is known to be highly dependent on the sample, and in fact, previous studies have also shown variations in the transition temperature^9,15–17^. In contrast to measurements such as magnetization, which observe the average structure of the entire sample, the phase boundaries in the present study were determined from the intensity and half-width of the diffraction peaks corresponding to the magnetic modulation, which is possible to extract only the spin-modulated structural information of the chiral magnet.

Temperature dependence of the full-width-half-maximum (FWHM) of fundamental magnetic diffraction ($I_1$), and the integrated intensities of magnetic diffractions ($I_1$ and $I_2/I_1$) are plotted in Fig. 4b,c, respectively. Herein, the magnetic transition temperature $T_{\mathrm{N}} = 119.5\ \hbox {K}$, was determined by the temperature dependence of the FWHM, where the peak width along the $\parallel q$ (*z* direction) started to broaden. Below $T_{\mathrm{N}}$, the helical state appeared only near the zero magnetic field, whereas the 2nd order magnetic diffraction emerged with the application of magnetic field, corresponding to the CSL state, as shown in the temperature dependence of the 2nd order magnetic diffraction ($I_2/I_1$) in Fig. 4c. Moreover, the theoretically expected $I_2/I_1$ value at $q/q_0=0.9$ was approximately 0.055 as indicated by the broken line, from which the observed intensities deviated with an increase in temperature and finally disappeared at $T_{\mathrm{N}}$. The behavior indicates the CSL was formed until $T_{\mathrm{N}}$ even though its structure was distorted from the theoretical one near $T_N$. In contrast, the fundamental magnetic diffraction was observable even above $T_{\mathrm{N}}$ and finally disappeared at $T_{SRO}\sim 121.5\ \hbox {K}$. In the temperature range $T_{\mathrm{N}}<T<T_{SRO}$, while the FWHM for $\parallel q$ became broader with increasing temperature, that for $\perp q$ (*y* direction) kept its low temperature values. These results suggest that the short-range order (SRO) of helical magnetic state survives even above $T_{\mathrm{N}}$. In a calculation study for $\hbox {CrNb}_3\hbox {S}_6$, the in-plane magnetic interaction $J_\perp$ is estimated to be stronger than $J_\parallel$^18^. This indicates the strong ferromagnetic interaction within the *ab* plane, which satisfies the longer correlation length for $\perp q$ direction as observed. Since the 2nd order diffraction was not observable in the region, *i*.*e*. $T_{\mathrm{N}}<T<T_{SRO}$, direct transitions from Hx to FFM state would occur at $H_c$ without forming the CSL. A similar magnetic state near $T_N$ has also been reported in the magnetization measurements and is assigned to be the CSL state^16^. However, our diffraction experiments have not observed any higher-order reflections that are evidence for forming CSL, thus we can identify it as a helical or a fan-like magnetic state with the short-range order.

**Figure 5:**
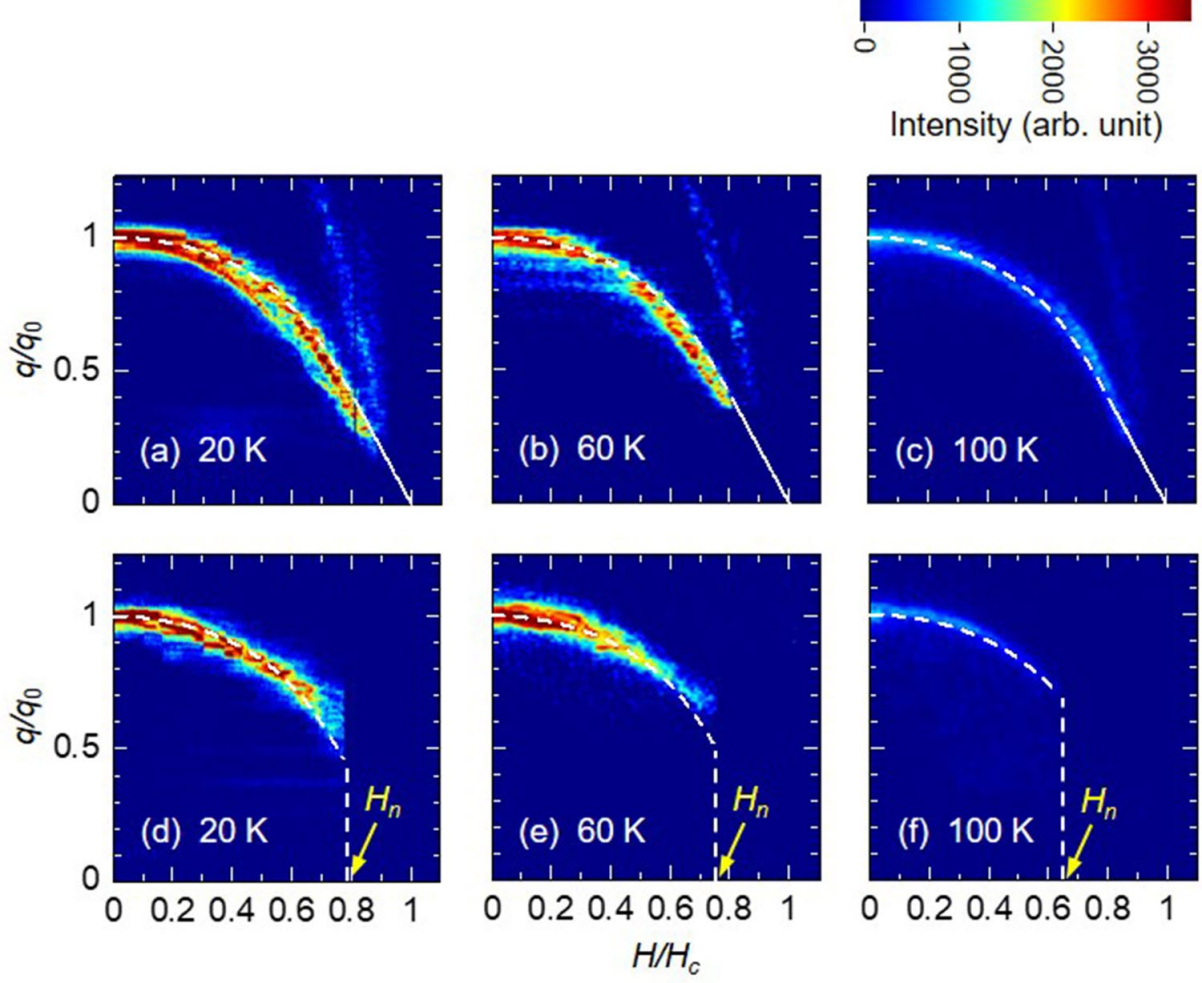
Hysteresis behavior between ferromagnetic and chiral soliton lattice state. Contour plot of magnetic diffraction line profile in $q>0$ region during (**a**–**c**) increasing and (**d**–**f**) decreasing magnetic field process, measured at (**a**,**d**) 20, (**b**,**e**) 60, and (**c**,**f**) 100 K. Magnetic modulations are normalized by $q_0$. White lines indicate the theoretically calculated curves. Straight lines in the higher *q* region in (**a**–**c**) correspond to the 2nd order magnetic diffraction from the chiral soliton lattice. $H_n$ indicates the value of the magnetic field at which the magnetic reflection starts to be observed in the course of decreasing the magnetic field.

Respective nucleation and annihilation processes of chiral solitons during decreasing and increasing magnetic field are crucial and unsolved issues for CSL in $\hbox {CrNb}_3\hbox {S}_6$. We unveiled these processes by showing contour plots of the diffraction profiles at several temperatures in Fig. 5, both in the processes of increasing and decreasing the magnetic-field. Here, note that the magnetic modulation vector *q* and magnetic field *H* were normalized by $q_0$ and $H_c$, respectively. Apparently, the behavior of $q/q_0$ in the increasing magnetic-field process, i.e., the soliton annihilation process, was well reproduced as the theoretical curve, as depicted in the white dash line in Fig. 5a–c. The behavior of the 2nd order diffraction were also recognizable as straight lines in the higher *q* region of Fig. 5a–c. The fundamental magnetic diffraction could be observed continuously up to near the transition magnetic field ($H/H_c=1$) and its intensity gradually decreased. In contrast, in the process of decreasing the magnetic field from the FFM state, i.e. the soliton nucleation process, the soliton was discontinuously generated from the magnetic field lower than the transition magnetic field ($H_C$). Here, the magnetic field value was denoted by $H_n$. Below $H_n$, the changes of $q/q_0$ against magnetic field were on the theoretical curves. Those results indicate that, while the number of chiral solitons gradually decreases with higher magnetic field, a certain number of chiral solitons occur simultaneously at $H_n$, and then the number of chiral solitons gradually increases below $H_n$ with lower magnetic field. Such hysteresis regions were seen at all measurement temperatures with $H_n$ becoming higher as the temperature going down^19^. In ferromagnetic materials, a considerable increase of the coercive field with a lowering temperature has usually been reported^20^. The coercivity, which can be related to a domain wall pinning, shows an exponential decay with temperature^21^. It indicates that the hysteresis of the chiral soliton cannot be explained by such a pinning effect model discussed in the ferromagnetic materials.

**Figure 6:**
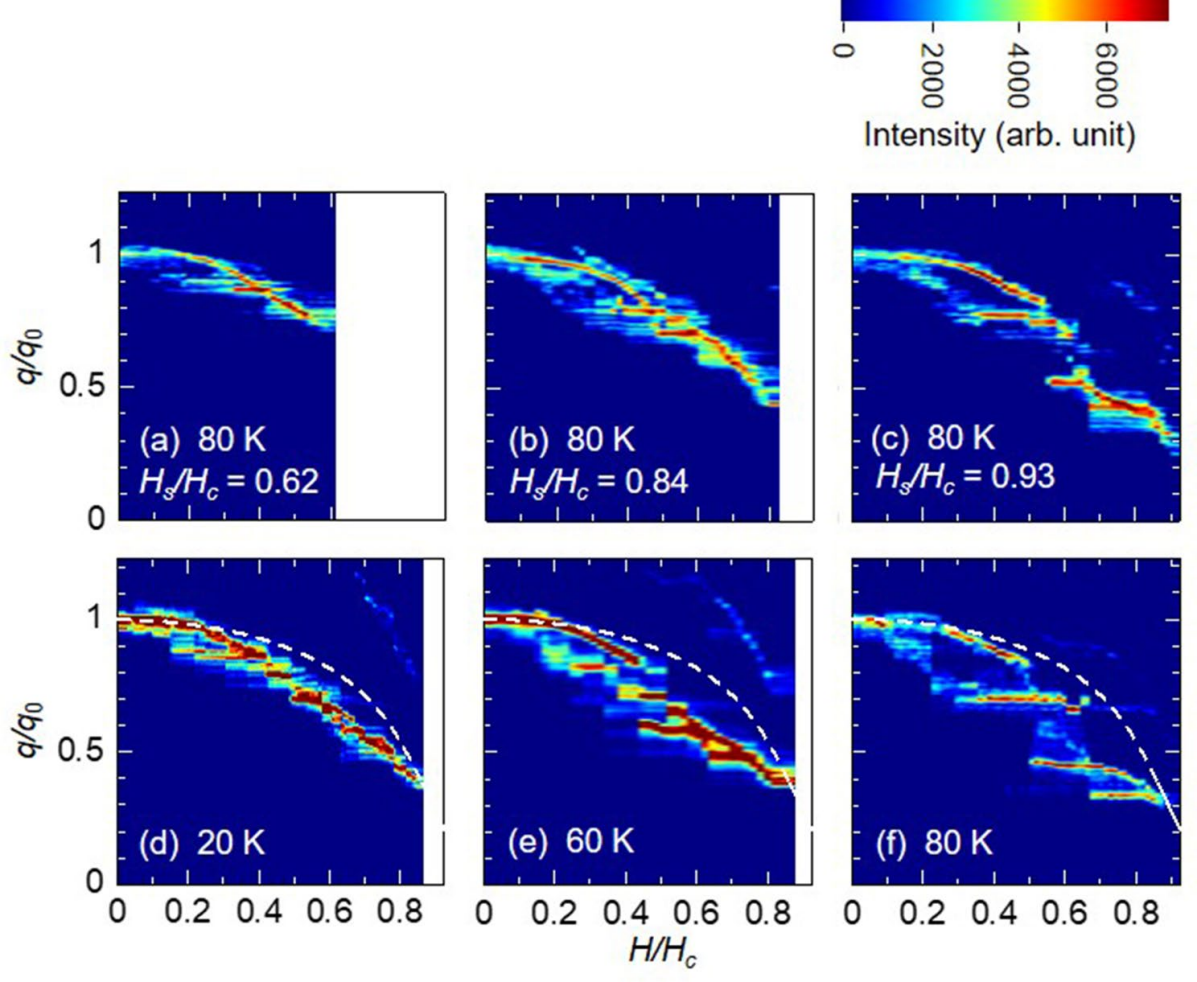
Hysteresis behavior between ferromagnetic and chiral soliton lattice state. (**a**–**c**) The chiral soliton creation process with varying initial fields ($H_s/H_c$). After magnetic field is increased from zero to each $H_s$, the change in propagation vector $q/q_0$ is measured from each $H_s$ with decreasing magnetic field. White areas indicate that the measurements are not made. (**d**–**f**) Temperature dependence of the chiral soliton creation process with decreasing magnetic field. The initial fields are fixed at $H_s/H_c=0.90$. White dash lines denote the theoretical curve.

A more complicated chiral soliton generation process was also observed in the present study. The $q/q_0$ dependence on magnetic field $H/H_c$ is depicted in Fig. 6a–c as well as in Fig. 5. Here, the different points were the initial states where the magnetic field started decreasing. More specifically in Fig. 5, the magnetic field started to decrease from the FFM state, whereas in Fig. 6a–c, from the state where the chiral soliton still remained, that is, Fig. 6a–c show the respective chiral soliton nucleation process that started from magnetic field values of $H_s/H_c=0.62$, 0.84, and 0.93, after the magnetic field was increased from zero to each $H_s/H_c$ values. Evidently, here the formation process of chiral solitons greatly differed relative to the initial state. Starting from a low $H_s$, *i.e*. $H_s/H_c =0.62$, where a large amount of chiral solitons remained, the number of chiral soliton gradually increased according to the theoretical curve. However, when $H_s/H_c = 0.93$ where the number of chiral soliton might be quarter of that at the zero field as shown in Fig. 6c, the $q/q_0$ behavior greatly deviated from the theoretical curve; it exhibits a step-wise and a plateau-like behavior. It can be seen that whether the nucleation process of chiral solitons matches the theoretical value depends strongly on the initial state. In X-ray magnetic circular dichroism (XMCD) measurements, a similar behavior, in which the shape of minor loop magnetization curve depends on the initial state, has been also reported for the CSL in $\hbox {CrNb}_3\hbox {S}_6$^13^. The temperature dependence of chiral soliton nucleation process starting from $H_s/H_c =0.9$ is shown in Fig. 6d–f. Although the step-wise and plateau-like behavior were present at all measurement temperatures, the height of step and the width of plateau region became larger as the temperature increased. Similarly, in the case of $H_n$ in Fig. 5, we observed highly distinct different behaviors in the nucleation and annihilation of chiral solitons with higher temperatures, which indicate the possibility of the thermal fluctuation largely affecting the formation of chiral solitons.

## Discussion

The nucleation and annihilation of chiral soliton deserve a discussion emphasis based on its topological stability and the thermal fluctuation. In the present diffraction experiment, we showed a clear separation between the helical and chiral soliton by the behavior of higher order magnetic reflections, which demonstrated good agreement with the theoretical prediction based on the monoaxial sine-Gordon model. During the process of increasing the magnetic field, i.e. destroying the chiral solitons, we showed the relative consistency of the gradual change in $q/q_0$ with the theoretical curve. Clearly, the potential barrier related to the topological protection must be overcome to annihilate each chiral soliton. Some possible destroying paths include an ejection to outside from the sample’s boundary, a chiral soliton collapse, and a chiral soliton destruction mediated by a singularity^22^. In a high density chiral soliton state, where the Zeeman energy acts for the soliton repulsion to push the soliton to the outside, the lowest energy path might be the boundaries of the sample^23^. As discussed in the context of the skyrmion system, the topological protection was not robust at an edge of a finite size sample^24^. Given a finite temperature, thermally activated magnon bounded to the edge of the sample would condense at a finite momentum, making the edge locally unstable^25^ so as to assist the chiral soliton jump over the potential barrier of the topological protection and to unwind the spin kink, consequently driving out the chiral solitons. Considering the process of disappearance of the chiral soliton at the boundary with lack of topological protection, we find it plausible that the increasing magnetic field process agrees well with such theoretical curve that does not consider the topological barrier for destroying the soliton.

In the case of decreasing the magnetic field, the microfabrication sample exhibits large hysteresis, step-wise, and plateau-like behavior in the magnetic propagation vector $q/q_0$, describing a large deviation from the theoretical curve. These results are very indicative that the potential barrier of topological protection is affected when chiral solitons are generated. The origin of hysteresis upon the phase transition should be the metastable state due to the topological stability, i.e., the topological metastability. Indeed, a similar discontinuous behavior has been reported as with various measurement methods, such as magnetization, magnetoresistance (MR)^9^, ferromagnetic resonance, and magnetic torque measurement. Recently, the discontinuous behavior in the MR measurement has been discussed based on a surface barrier for isolated chiral soliton to enter a sample^23^. Thus, it was theoretically addressed that $H_{jump}$ where a discontinuous jump occurs in the MR can be identified as a characteristic field of $H_b\sim 0.4H_c$, within which the chiral soliton may possibly enter the sample due to the disappearance of the surface barrier^23^. A similar phenomenon has been also discussed in the two-dimensional skyrmion system^25^, in which edge instabilities due to magnon condensation crucially triggered the formation of the chiral soliton^26^. We believe that a discussion on the topological metastability mentioned herein can qualitatively explain the hysteresis behavior observed in the present experiment, as shown in Fig. 5. However, the magnetic field value of $H_n/H_c\sim$ 0.6–0.8 observed in the magnetic diffraction, which should correspond to $H_{jump}$ in the MR measurement, were higher than the theoretical value $H_b\sim 0.4 H_c$, likewise demonstrating temperature dependence. Thus, considering other instabilities is imperative in overcoming the topological protection due to irregularities of sample edge and thickness and/or that of randomness (pinning center), resulting in inhomogeneous magnetic field within the sample^27^.

Furthermore, as the topological defects cannot be annihilated or created inside the sample without overcoming the larger energy barrier due to the topological protection, they must be robust against thermal fluctuation and disturbances. With no account of quantum tunneling, the transition rate of creating or annihilating a single chiral soliton should obey the Arrhenius law $f = f_0 \exp (-\Delta /k_BT)$ with $f_0$ being the attempt frequency, $\Delta$ the topological protection barrier, *T* the temperature and $k_B$ the Boltzmann constant^22^. If the energy barrier $\Delta$ is sufficiently larger than the thermal fluctuation $k_BT$, then the transition rate becomes sufficiently small, so that topological defects can exist stably in the sample. As a discernible consequence, lowering the temperature should suppress the creation and annihilation of the chiral solitons, because thermal fluctuation would be unable to overcome the potential barrier. In the same manner, heating sample would activate the transition rate. From these estimations, we could presume that in the magnetic field sweep process, the change in number of chiral solitons would occur smoothly at higher temperatures, whereas stagnation and discontinuous change are likely to occur at lower temperatures. However, the experimental results showed totally different behaviors from these expectations; $H_n$ is higher as the temperature rises (Fig. 5), and the stepwise and the plateau-like behavior appears more clearly at higher temperatures (Fig. 6). The situation seems that the thermal fluctuation suppresses the change of the topological number and assists the topological protection. This point cannot be explained simply by treating the chiral soliton as the topological defect (the pseudo particle). Rather, the thermal fluctuation on the internal degree of freedom of the chiral soliton should be considered.

**Figure 7:**
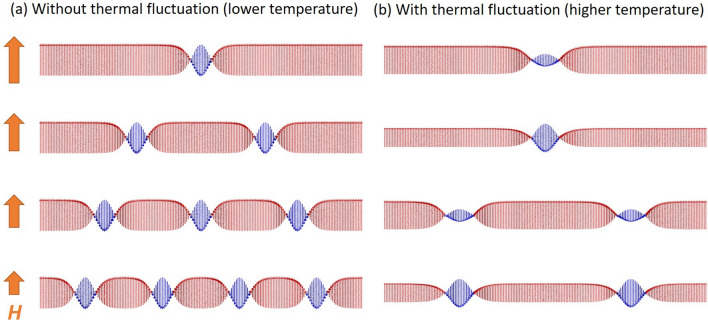
Variation of magnetic structure with decreasing magnetic field: schematic pictures for the magnetic structure of chiral solitons with decreasing magnetic field (*B*) at (**a**) a lower temperature and (**b**) a higher temperature. Because thermal fluctuation would be strongly suppressed at the lower temperature, the number of chiral soliton must increase one by one during the magnetic field decreasing process to match the theoretical model. On the opposite, at the higher temperature, the thermal fluctuation would suppress the increment of chiral soliton. This is because the thermal fluctuation causes the thermal average of spins to shrink compared to its saturation value.

In a finite system, the maximum number of chiral soliton $n_{max}$, i.e. topological number, is determined by the system size. In the decreasing magnetic field process, the topological number *n* changed from 0 (the FFM state) to $n_{max}$ (the Hx state). The energy level for each number of chiral soliton can be calculated by the sine-Gordon model with fixed spins on both ends of sample^28^. The critical fields for transition from one topological number to another with a larger number are determined the energy crossing points. The energy barrier to change the topological number, relating the topological protection, depends on the magnetic field and the topological number itself^28^. Based on the discussion of hysteresis, the existence of the surface barrier (or the topological metastability) would result to the topological number change at greater than 1. Transitions from the *n*-th topological sector to the *m*-th one will occur at the magnetic field where the energy difference between these two states ($\Delta E= E_n-E_m$) should exceed the surface barrier. As the thermal fluctuation increases, the deformation of a single chiral soliton becomes possible, thus the energy change of the CSL against the change of the magnetic field becomes gentler. In other words, at one magnetic field, any energy difference between the two states of a different topological number becomes smaller with an increasing thermal fluctuation. Therefore, to exceed the surface barrier, more energy difference by magnetic field is required at higher temperature, which should result in the discontinuous and plateau-like behavior in the chiral soliton number in the decreasing magnetic field process. Such behavior can also be explained with a simple model shown in Fig. 7. At lower magnetic field, the ferromagnetic region becomes unstable and another chiral soliton is generated to obtain an energy gain from the DM interaction. The number of chiral soliton would increase one by one during the magnetic field decreasing process to match the theoretical model (Fig. 7a). Enhancing thermal fluctuation at a higher temperature would cause the thermal average of spins ($\langle S \rangle$) to shrink relative to its saturation value at the lower temperature; $\langle S \rangle$ can be flexibly changed by the effective magnetic field (Fig. 7b). At certain high magnetic fields, $\langle S \rangle$ inside chiral solitons would be reduced compared to $\langle S \rangle$ in the ferromagnetic region. Thus, lowering the magnetic field would cause $\langle S \rangle$ in the ferromagnetic region to conversely shrink, while $\langle S \rangle$ in the chiral soliton would be enhanced. At higher temperature, such changes in the magnetic structure become possible instead of the increment of chiral solitons. Another possibility of magnetic structure change is deformation of chiral soliton from the theoretically expected shape such as an enlargement of the width. If such deformations of the magnetic structure are no longer possible, one or more chiral soliton will be added. Thus, the observed step-wise and the plateau-like behavior can be interpreted by such magnetic structure change during the decreasing magnetic field processes. Moreover, we could infer that the influence on the metastable state by the magnetic field change is reduced by the thermal fluctuation and the change of the topological number is suppressed, thus the thermal fluctuation should assist the topological stability in the present system. Conversely, at low temperatures, thermal fluctuations are suppressed, and thus smooth topological number changes of CSL are realized. The effects of thermal fluctuation on the topological protection have been also actively discussed in the skyrmion system^29,30^. The relevance of such effects, i.e. the difference in dimensionality, is a very interesting topic for a future work.

## Conclusion

For the present study, we clarified that the magnetic-field dependence of chiral soliton lattice agrees well with the theoretically predicted magnetic structure based on the monoaxial spin Hamiltonian from the observed intensity ratio of higher harmonic magnetic diffraction. Some irreversible behaviors upon magnetic-field responses were observed, not only describing the continuous deformation of the soliton lattice in the increasing magnetic-field process but also the discontinuous change. The experimental method we employed was able to clarify the accurate modulation of the magnetic structure; thus, it is applicable for the observation of intriguing dynamical phenomena proposed herein. Moreover, we elucidated that the soliton creation process is sensitive to the thermal fluctuation, and that subsequently, the changing of topological number in 1D chiral magnetic system is suppressed by the thermal fluctuation of spins.

## Methods

### Sample fabrication

We used the chemical transport method to grow a single crystal of $\hbox {CrNb}_3\hbox {S}_6$^8^ with a volume of $\sim 0.01\ \hbox {mm}^3$, and had it characterized via magnetization measurements. A thin plate with a thickness of $\sim 120\ \hbox {nm}$ was prepared by the focused ion beam (FIB) thinning method (SMI3200; Seiko Instruments Inc., Japan) for the small-angle RSXS observation. The attenuation length of $\hbox {CrNb}_3\hbox {S}_6$ was estimated at approximately 150 nm at the Cr $L_3$-edge. We affixed the sample with carbon contacts on a $\hbox {Si}_3\hbox {N}_4$ substrate with a square hole of $10 \times 10\ \mu \hbox {m}^2$. No clear inhomogeneity was observed in the FIB fabricated sample by the scanning electron microscopy.

### Resonant soft X-ray small-angle scattering

Small-angle RSXS measurements were carried out at a soft X-ray beamline BL-16A in Photon Factory, KEK, Japan, that was equipped with a vacuum chamber having a background pressure of $1 \times 10^{-8}$ Torr (Fig. 1d)^12,31^. The incident soft X-rays were tuned to be circularly polarized, i.e., left-handed circularly polarization (LCP) and right-handed circularly polarization (RCP) with a beam size of 0.6 mm (H) $\times 0.4\ \hbox {mm}$ (V). An in-vacuum CCD camera ($2048 \times 2048\ \hbox {pixels}$, Teledyne Princeton Instruments), positioned downstream of the sample, was used to record the RSXS intensity.
